# Whole Genome Comparisons Suggest Random Distribution of *Mycobacterium ulcerans* Genotypes in a Buruli Ulcer Endemic Region of Ghana

**DOI:** 10.1371/journal.pntd.0003681

**Published:** 2015-03-31

**Authors:** Anthony S. Ablordey, Koen Vandelannoote, Isaac A. Frimpong, Evans K. Ahortor, Nana Ama Amissah, Miriam Eddyani, Lies Durnez, Françoise Portaels, Bouke C. de Jong, Herwig Leirs, Jessica L. Porter, Kirstie M. Mangas, Margaret M. C. Lam, Andrew Buultjens, Torsten Seemann, Nicholas J. Tobias, Timothy P. Stinear

**Affiliations:** 1 Department of Bacteriology, Noguchi Memorial Institute for Medical Research, University of Ghana, Accra, Ghana; 2 Department of Biomedical Sciences, Institute of Tropical Medicine, Antwerp, Belgium; 3 Department of Animal Biology and Conservation Science, University of Ghana, Accra, Ghana; 4 Department of Biology, University of Antwerp, Antwerp, Belgium; 5 Department of Microbiology and Immunology, University of Melbourne, Parkville, Australia; 6 Life Sciences Computation Centre, Victorian Life Sciences Computation Initiative, Carlton, Victoria, Australia; Fondation Raoul Follereau, FRANCE

## Abstract

Efforts to control the spread of Buruli ulcer – an emerging ulcerative skin infection caused by *Mycobacterium ulcerans* - have been hampered by our poor understanding of reservoirs and transmission. To help address this issue, we compared whole genomes from 18 clinical *M*. *ulcerans* isolates from a 30km^2^ region within the Asante Akim North District, Ashanti region, Ghana, with 15 other *M*. *ulcerans* isolates from elsewhere in Ghana and the surrounding countries of Ivory Coast, Togo, Benin and Nigeria. Contrary to our expectations of finding minor DNA sequence variations among isolates representing a single *M*. *ulcerans* circulating genotype, we found instead two distinct genotypes. One genotype was closely related to isolates from neighbouring regions of Amansie West and Densu, consistent with the predicted local endemic clone, but the second genotype (separated by 138 single nucleotide polymorphisms [SNPs] from other Ghanaian strains) most closely matched *M*. *ulcerans* from Nigeria, suggesting another introduction of *M*. *ulcerans* to Ghana, perhaps from that country. Both the exotic genotype and the local Ghanaian genotype displayed highly restricted intra-strain genetic variation, with less than 50 SNP differences across a 5.2Mbp core genome within each genotype. Interestingly, there was no discernible spatial clustering of genotypes at the local village scale. Interviews revealed no obvious epidemiological links among BU patients who had been infected with identical *M*. *ulcerans* genotypes but lived in geographically separate villages. We conclude that *M*. *ulcerans* is spread widely across the region, with multiple genotypes present in any one area. These data give us new perspectives on the behaviour of possible reservoirs and subsequent transmission mechanisms of *M*. *ulcerans*. These observations also show for the first time that *M*. *ulcerans* can be mobilized, introduced to a new area and then spread within a population. Potential reservoirs of *M*. *ulcerans* thus might include humans, or perhaps *M*. *ulcerans*-infected animals such as livestock that move regularly between countries.

## Author Summary

In this study we use the power of whole genome sequence comparisons to track the spread of *Mycobacterium ulcerans*, the causative agent of Buruli ulcer, through several villages in the Ashanti region of Ghana, providing new insights on the behaviour of this enigmatic and emerging pathogen.

## Introduction

Buruli ulcer (BU) is a neglected tropical disease caused by infection with *Mycobacterium ulcerans*. Each year 5000–6000 cases are reported from 15 of the 33 countries where BU cases have been reported, predominantly from rural regions across West and Central Africa [1]. The disease involves subcutaneous tissue and has several manifestations but necrotic skin ulcers are a common presentation, caused by the proliferation of bacteria beneath the dermis by virtue of a secreted bioactive lipid called mycolactone [2]. The role of mycolactone in the natural ecology of *M*. *ulcerans* is not understood, but it has been shown to possess several specific activities against mammalian cells from activating actin polymerization, blocking secreted protein translocation, to interacting with neuronal angiotensin type II receptors causing hypoesthesia [3–5]. These collective biological activities of mycolactone, while diverse, might collectively help explain the tissue destruction, lack of inflammation, and painlessness associated with BU. BU is rarely fatal and early diagnosis followed by combined antibiotic therapy (rifampicin and streptomycin) is key to preventing complications that can arise from severe skin ulceration [6].

Epidemiological studies frequently link BU occurrence with low-lying and wetland areas and human-to-human transmission seems rare, suggesting an environmental source of the mycobacterium [7–23]. Frustratingly however, the environmental reservoir(s) and mode(s) of transmission of *M*. *ulcerans* remain unknown. *M*. *ulcerans* has the genomic signature of a niche-adapted mycobacterium, indicating that it is unlikely to be found free-living in diverse aquatic (or other) environments, but more likely in close association with a host organism. In south eastern Australia, native marsupials have been identified as both susceptible hosts and reservoirs of *M*. *ulcerans*, with high numbers of the bacteria shed in the feces of infected animals. Mosquitoes have also been found to harbor the bacteria in this region and a zoonotic model of disease transmission has been proposed involving possums, biting insects and humans [24–26]. No such animal reservoir has yet been identified in African BU endemic areas and studies of BU lesion distribution are thought not consistent with mosquito biting patterns [22,27]. On the other hand, case-control studies in Cameroon have shown that bed nets are protective, supporting a role for insects in transmission [28].

A feature of *M*. *ulcerans* is the close correlation between genotype and the geographic origin of a strain, but its restricted genetic diversity has limited the application of traditional molecular epidemiological methods such as VNTR-typing to discriminate between isolates at the village or even regional scales. The advent of low cost genomics has opened up new possibilities to explore and track the movement and spread of this pathogen within communities [29,30].

Agogo is the principal town of 30,000 inhabitants in the Asante Akim North (AAN) district within the Ashanti region of Ghana and BU has been reported in about half of the sixty-four communities in this district since mid-1975 [10]. The AAN district covers an area of 650 km^2^ in the forest belt of Ghana and it is the third most endemic district in Ghana [31]. Five of the communities (Ananekrom, Serebouso, Nshyieso, Serebuoso and Dukusen) in this district are among the communities reported with the highest burden of the disease in Ghana [31]. About 120 laboratory-confirmed new cases are reported annually in this district [31]. Subsistence farming and petty trading are the principal occupations of inhabitants of these endemic communities. People generally live in simple dwellings constructed from local materials. Houses are often close together with 3–5 households in a compound. Many inhabitants raise animals such as goats, sheep, and pigs in the immediate vicinity of their houses. Farming is the main occupation with some people engaged in fishing and petty trading. Farms may be distant, ranging 5–20 km from a given domicile. Fishing is usually undertaken close to home. Water sources are of two types. Water for drinking and cooking is usually fetched from bore holes fitted with mechanical pumps, within or near a village. Water for bathing and domestic chores such as washing of clothes is drawn from local natural water sources (rivers, streams, ponds). These natural sources are usually no more than 500 metres from a given village.

In this study we sequenced and compared the genomes of 18 *M*. *ulcerans* isolates obtained from 10 BU endemic villages in the AAN district and uncovered genetic evidence supporting the introduction of a foreign clone of *M*. *ulcerans* to this region. This observation indicates that *M*. *ulcerans* can be mobilized and spread throughout a region, indicating that reservoirs of the bacterium are themselves potentially highly mobile.

## Methods

### Ethics statement

*M*. *ulcerans* isolates were obtained from BU diagnostic samples, collected as part of routine laboratory diagnosis. Ethical approval to interview patients and use bacterial isolates resulting from diagnostic specimens for research was obtained from the ethical review board of the Noguchi Memorial Institute for Medical Research, University of Ghana, Legon, Accra, Ghana (FWA 00001824), with written informed consent obtained from all adult patients or the parents/guardians of the participating children.

### Study site and case reporting

The study was carried out in ten endemic villages including Ananekrom, Nshyieso, Serebouso, Dukusen, Afreserie, Afreserie OK, Baama, Nysonyameye, Kwame Addo and Bebuso, in the Asante Akim North (AAN) district of Ghana (Table 1). These are small villages and hamlets, 5 to 10 km from each other with populations between 120–1500 inhabitants. Ananekrom is the largest of these communities and is the closest (15 km) to the district capital, Agogo. An asphalt road connects Agogo to Ananekrom, Dukusen and Afriserie, while the other communities are located off this main road and are connected to each other by unmade roads and foot-tracks. A community health centre Ananefromh (near Ananekrom) is usually the first point of call for patients seeking medical treatment. Patients suspected of having BU are referred to the Agogo Presbyterian Hospital for diagnosis and treatment. Patient information including name and place of residence were obtained from hospital records and patients were visited in their homes for more detailed interviews that included questions about possible travel to other BU endemic areas outside the AAN district. GPS coordinates in the vicinity of each patient’s residence were recorded in order to map the spatial distribution of cases in the villages, based on the assumption that the patient acquired their infection near their domicile.

**Table 1:** *M*. *ulcerans* isolate and DNA sequencing information.

No.	Strain ID^1^	Isolation date	Patient Age (years)	Patient Gender	Village/Genotype	District/ Country	Location^2^	Seq Plat.	Avg cov.	ENA	Ref.
1	S15	02/02/2010	5	M	Ananekrom/ Agogo-1	Ashanti/Ghana	6.91481, -1.01658	SE PGM	106		This study
2	S43	09/05/2010	13	F	Ananekrom/ Agogo-2	Ashanti /Ghana	6.91481, -1.01658	SE PGM	120		This study
3	S38	23/06/2010	12	M	Serebuso/ Agogo-1	Ashanti /Ghana	6.94838, -1.02373	SE PGM	104		This study
4	F64	08/09/2010	3	M	Nsonyameye/ Agogo-1	Ashanti /Ghana	6.93744, -0.96464	SE PGM	99		This study
5	F70	15/09/2010	32	F	Baama/ Agogo-1	Ashanti /Ghana	6.96810, -0.94013	SE PGM	61		This study
6	1510 (F79)	22/09/2010	28	F	Bebuso/ Agogo-2	Ashanti /Ghana	6.88909, -0.97209	PE Illumina	81		This study
7	F75	07/10/2010	18	F	Afriserie OK/ Agogo-1	Ashanti /Ghana	7.01992, -0.92325	SE PGM	68		This study
8	F85	20/10/2010	28	F	Nshyieso/ Agogo-2	Ashanti /Ghana	6.95910, -1.01860	SE PGM	71		This study
9	S77	24/11/2010	3	F	Serebuso/ Agogo-2	Ashanti /Ghana	6.94838, -1.02373	SE PGM	83		This study
10	2610 (F92)	24/12/2010	14	F	Nsonyameye/ Agogo-1	Ashanti /Ghana	6.93744, -0.96464	PE Illumina	67		This study
11	F13	02/02/2011	8	F	Bebosu Ado/ Agogo-1	Ashanti /Ghana	6.88909, -0.97209	SE PGM	92		This study
12	F65	02/09/2011	28	F	Ananekrom/ Agogo-2	Ashanti /Ghana	6.91481, -1.01658	SE PGM	89		This study
13	F74	21/09/2011	38	M	Dukusen/ Agogo-2	Ashanti /Ghana	6.97683, -0.98278	SE PGM	46		This study
14	S72	12/12/2011	11	M	Afriserie/ Agogo-1	Ashanti /Ghana	7.01992, -0.92325	SE PGM	128		This study
15	612 (F36)	18/07/2012	37	M	Ananekrom/ Agogo-1	Ashanti /Ghana	6.91481, -1.01658	PE Illumina	97		This study
16	212 (F3)	08/02/2012	11	F	Serebuso/ Agogo-2	Ashanti /Ghana	6.94838, -1.02373	PE Illumina	62		This study
17	712 (S24)	02/08/2012	40	F	Wenamda/ Agogo-2	Volta/Ghana	7.07738, 0.092016	PE Illumina	214		This study
18	412 (F37)	02/08/2012	12	M	Ananekrom/ Agogo-1	Ashanti /Ghana	6.91481, -1.01658	PE Illumina	162		This study
19	IC21	07/2000			Bondoukou	Cote d’Ivoire	8.03333, -2.8000	SE PGM	79		This study
20	IC38	07/2000			Dimbokro	Cote d’Ivoire	6.64445, -4.70540	SE PGM	63		This study
21	ITM000909	2000			Tchekpo Deve	Maritime/Togo	6.48434, 1.369718	SE PGM	45		[45]
22	ITM991591	1999			Anagali	Maritime/Togo	6.48434, 1.369718	SE PGM	55		[45]
23	ITM102686	2010		M	Ibadan	Oyo State/Nigeria	7.50194, 3.982327	SE PGM	51		[45]
24	ITM5151	1971				Maniema/DRC^3^	-4.18655, 26.43937	SE PGM	51		[45]
25	NM14.01	2001				Densu/Ghana	5.72532, -0.31075	PE Illumina	111		[42]
26	NM43.02	2002				Densu/Ghana	5.69881, -0.38597	PE Illumina	112		[42]
27	NM49.02	2002				Densu/Ghana	5.70209, -0.29818	PE Illumina	114		[42]
28	NM54.02	2002				Densu/Ghana	5.6568, -0.32419	PE Illumina	73		[42]
29	NM33.04	2004				Amansie West/Ghana	6.68712, -1.62197	PE Illumina	84		[42]
30	Agy99^4^	08/1999	5	F	ulcer	Amansie West/Ghana	6.68712, -1.62197	Sanger			[41]
31	ITM980535	1998	12	F	Djigbé	Atlantique/Benin	6.885076, 2.362017	PE Illumina	257		[42]
32	ITM000945	2000	21	F	Hwegoudo	Atlantique/Benin	6.7234, 2.377314	PE Illumina	231		[42]
33	ITM001506	2000	6	F	Wokon	Zou/Benin	7.099851, 2.464233	PE Illumina	233		[42]

Notes:

^1^parentheses indicate alternate strain code,

^2^expressed as latitude and longitude,

^3^Democratic Republic of the Congo,

^4^reference genome

### Culture isolation and identification of *M*. *ulcerans* from patients

The isolates examined in this study are listed in Table 1 and were recovered from fine needle aspirates (FNA) or swabs, obtained from pre-ulcerative lesions and ulcers respectively. Specimens were stored in transport medium and PBS and transported in cool boxes to the Noguchi Memorial Institute for Medical Research (NMIMR) for diagnosis [32,33]. Tubes containing swabs were vortexed in 3 ml of transport medium for 30 sec and the swabs removed. A volume of 250μl of the transport medium from either specimen type was transferred into 1.5 ml microfuge tubes and decontaminated using the oxalic acid method as previously described [34]. The pellets were resuspended in 100 μl phosphate buffered-saline (PBS) and 100 μl volume of the decontaminated sample was inoculated onto Löwenstein Jensen (LJ) slopes and incubated at 33°C. The cultures were observed weekly for growth. Suspected *M*. *ulcerans* colonies were harvested and DNA extracted as described above [35]. The DNA extract was tested with the IS*2404* PCR for the identification of *M*. *ulcerans* [36]. Colonies positive for IS*2404* were suspended in 1 ml of Middlebrook 7H9 broth and stored at -80°C. All 18 bacterial samples analyzed were selected from this stored collection and were subcultured on LJ medium and DNA for whole genome sequencing was extracted from resulting growth as described [35]. The isolation date refers to the date when colonies became visible on LJ medium following primary cultivation.

### Genome sequencing and analysis

DNA sequencing was performed using two methods. The Ion Torrent Personal Genome Machine was employed, with a 316 chip and 200bp single-end sequencing chemistry (Life Technologies). Genomic libraries for Ion Torrent sequencing were prepared using Ion Express, with size selection using the Pippin Prep (Sage Sciences) and emulsion PCR run using a One-Touch instrument (Life Technologies). The Illumina MiSeq was also used, with Nextera XP library preparation and 2x250 bp sequencing chemistry. Read data for the study isolates have been deposited in the European Nucleotide Archive (ENA) under accession ERA401876. Prior to further analysis, reads were filtered to remove those containing ambiguous base calls, any reads less than 50 nucleotides in length, and containing only homopolymers. All reads were furthermore trimmed removing residual ligated Nextera adaptors and low quality bases (less than Q10) at the 3' end. Resulting sequence Fastq sequence read files from either platform were subjected to read-mapping to the *M*. *ulcerans* Agy99 reference genome (Genbank accession number CP000325) using Bowtie2 v2.1.0 [37] with default parameters and consensus calling to identify SNPs (indels excluded) using Nesoni v0.109, a Python utility that uses the reads from each genome aligned to the core genome to construct a tally of putative differences at each nucleotide position (including substitutions, insertions, and deletions) (www.bioinformatics.net.au). Those positions in the Agy99 reference genome that were covered by at least 3 reads from every isolate defined a core genome. Note that the pMUM001 plasmid (required for mycolactone synthesis) was not included in the reference genome [38]. Testing of the plasmid sequences revealed less then 10 polymorphic sites among the genomes under investigation and the highly repetitive sequence structure of the mycolactone genes impaired unambiguous read-mapping. An unpaired t test with Welch’s correction was used to assess the differences between mean nucleotide pairwise identities for different groups of genomes. The null hypothesis (no difference between means) was rejected for p<0.01. The inputs for subsequent phylogenomic analyses were the nucleotide sequence alignments of the concatenated variable nucleotide positions for the core genome among all isolates. A maximum-likelihood (ML) phylogeny was inferred using RAxML v 7.2.8, with the GTR model of nucleotide substitution (plugin within Geneious v 8.0.4). We performed 1000 rapid pseudo-replicate bootstrap analyses to assess support for the ML phylogeny. We used Consensus-Tree-Builder (Geneious v8.0.4) to collapse nodes in the tree with bootstrap values below a set threshold of 70%. The resulting phylogenomic tree was exported in Newick format and visualized using FigTree v1.4.0 (tree.bio.ed.ac.uk/software/figtree). A haplotype network was derived using the median-joining algorithm as implemented in SplitsTree v4.13.1 [39,40]. A correction to the source attribution of the *M*. *ulcerans* Agy99 reference genome was also made in the course of this study, where it was realized that this isolate was actually obtained from a patient attending St Martin’s Hospital in Agroyesum (Amansie West) and not the Ga District Hospital as originally published [41] (K. Asiedu and J., Hayman pers comms), thereby explaining the inconsistent geographic clustering reported in previous molecular epidemiological studies [30,42,43].

## Results

### Genome sequence comparisons of 18 *M*. *ulcerans* isolates from Agogo region

**Fig 1:**
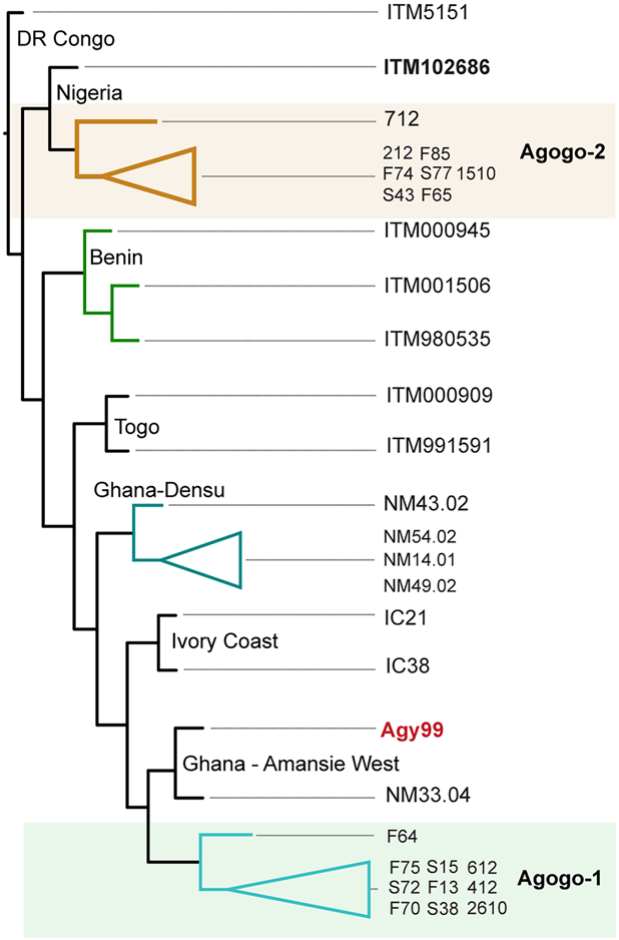
Genetic relationship among the 33 *M*. *ulcerans* isolates used in this study. A maximum-likelihood consensus phylogeny was inferred based on whole genome alignments of each of the isolates against the *M*. *ulcerans* Agy99 reference genome. The alignment file from pairwise comparisons of the resulting 320 variable nucleotide positions was used as input for RaxML. Nodes with less than 70% bootstrap support (1000 replicates) were collapsed.

Eighteen *M*. *ulcerans* isolates were randomly selected for whole genome sequencing. The isolates represented 20% (total of 92 isolates from 2010–2012) of all culture-confirmed BU cases referred to the Agogo Presbyterian Hospital between 2010 and 2012 (Table 1). There were no differences in colony phenotype or growth characteristics among the isolates. The DNA sequence reads for each genome were mapped to the *M*. *ulcerans* Agy99 reference sequence. Sequencing and read-mapping summary statistics are given in Table 1. In addition to the 18 Agogo isolates sequenced here, 15 other genomes (including some previously described) were included in comparisons making a total of 33 isolates (Table 1). These additional genomes were from *M*. *ulcerans* isolates in other regions of Ghana and from surrounding countries to provide appropriate genetic context for interpreting the diversity and evolution of *M*. *ulcerans* isolates from around Agogo. Read-mapping and SNP identification revealed 320 variable nucleotide positions across a 5.2Mb core genome for the 33 isolates. A phylogeny was inferred from this alignment, showing the clustering typical of *M*. *ulcerans* genotypes with geographic origin (Fig. 1). A separate SNP alignment was performed taking the genome sequences for only the 18 isolates from the Agogo region, and 10 of them (called Agogo-1) clustered with isolates from the neighboring district of Amansie West and also the Ivory Coast, the country which borders this region to the west (Fig. 1). This close relationship is indicative of a local clone that has spread and persisted within the greater region for some time. Unexpectedly however, this analysis also revealed the presence of a second distinct *M*. *ulcerans* genotype co-circulating with Agogo-1. This second genotype (called Agogo-2) was substantially more diverse from all other Ghanaian *M*. *ulcerans* genotypes (138 SNPs), suggesting the re-introduction of *M*. *ulcerans* to the Agogo region, potentially from a source outside Ghana (Fig. 1, S1 Table). The intra-genotype variation within either cluster was low. The mean nucleotide pairwise identity was 94.7% (SEM ± 0.4) for Agogo-1 versus 97.2% (SEM ± 0.4) for Agogo 2. The mean pairwise nucleotide identity was significantly lower for Agogo-2 genomes compared with Agogo-1 (*p*<0.001).

### A likely foreign origin for Agogo-2

To investigate the possible origin of the Agogo-2 isolates we compared SNP profiles among our panel of *M*. *ulcerans* genomes from across West and Central Africa. The closest match obtained was to isolate ITM102686, obtained from a patient originating from Ibadan, Nigeria, with 29 SNPs different when only this genome was compared to the Agogo-2 cluster. This close association may indicate that Nigeria was the source of the Agogo-2 cluster. Some circumspection is needed when interpreting these data, as only two *M*. *ulcerans* genomes were sampled from countries east of Benin. There is however a compelling patient history behind this isolate to support Nigeria as the correct origin. The Caucasian patient, a long-term resident in Ibadan and an employee of a non-government organisation, believes he was infected on an Ibadan golf course, when he was bitten by black biting flies (his description suggests they may have been moth flies [Psychodidae]) that began plaguing the course when ground works started adjacent to a lake on the course. The patient developed a painful ulcer on the site of the insect bites. A couple of months later he developed a second ulcer on an adjacent site on the same limb that was microbiologically diagnosed as a Buruli ulcer. This patient history, combined with the documented cases of BU in Ibadan, with cases occurring around the Ibadan University campus and other nearby institutions [44], support Ibadan as the likely origin of *M*. *ulcerans* isolate ITM102686.

### *M*. *ulcerans* genotype clustering breaks down at a local scale

**Fig 2:**
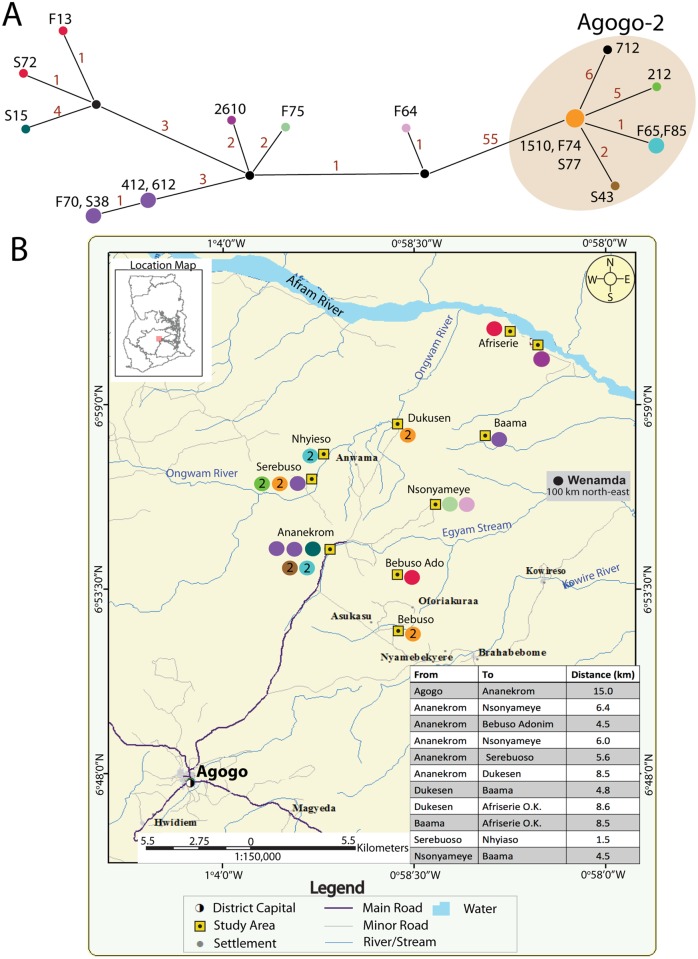
Micromolecular epidemiology of BU in the Asante Akim North District revealed by *M*. *ulcerans* whole genome sequence comparisons. (A) Median-joining network graph showing the genetic relationship between 18 *M*. *ulcerans* clinical isolates comprising the Agogo-1 and Agogo-2 genotypes (shaded), inferred from whole genome sequence alignments. Node sizes in the graph are proportional to the frequency of genotype occurrence and have been colour-coded accordingly. Edges are labelled in red with the number of mutational steps between each node. (B) Map of Asante Akim North District study area, showing the location of endemic villages and the origin of each of the 18 BU cases, with a coloured circle corresponding with the genotype displayed in the network graph in (A). The number “2” within some coloured circles indicates an Agogo-2 genotype.

We next explored the distribution of *M*. *ulcerans* genotypes in the Agogo region at the village scale and observed no obvious pattern or relationship between genotype, patient, strain and village (Fig. 2). There is complete intermixing of Agogo-1 and Agogo-2 clusters amongst the population. Median-joining-network analysis suggested the independent radiation of the two clusters throughout the region (Fig. 2). Furthermore, within either cluster there was a broad distribution of cluster subtypes across the region. For example, isolates F70 and S38 (Agogo-1) have identical SNP profiles but the patients came from Baama and Serebouso, villages separated by 10–15 km. Similarly isolates F74 and 1510 (Agogo-2), came from patients who live in two different villages (Fig. 2). Patient interviews did not identify any travel histories or other epidemiological links that might explain these distribution patterns. An 11-year old girl from Serebouso was the third child within her family to have BU (isolate 212, November 2012), eight years after two of her siblings had the disease. The family of this child lived very close to that of another BU patient, a 3 year-old infant (isolate S77, February 2010). Both isolates belonged to the Agogo-2 cluster but their genome sequence differed in nine nucleotide positions, a significant amount of genetic variation given that S77 shares a near identical genotype with F74 and 1510. Again, we could not identify any specific activity or travel history such as attending a common community event that was shared by Agogo-2 genotype patients. These data suggest that (i) the disease is acquired locally, (ii) multiple *M*. *ulcerans* genotypes are circulating simultaneously within the local region and (iii) a single clone can have the propensity to spread through a region. Further support for local acquisition of infection comes from observations of infants with no travel history with BU such as a locally-born 2-year old infant from Ananekrom identified over the time of this study.

## Discussion

The clonal population structure of *M*. *ulcerans* has made identifying and comparing genetic variation in isolates at anything less than a continental scale very difficult. Here we have used the high resolution afforded by comparative genomics to explore the molecular epidemiology of BU at the regional and village scale. Like recent studies using a single polymorphic genetic locus or whole genome sequence comparisons to assess *M*. *ulcerans* genetic diversity across a range of African countries, we found a highly significant relationship between the genotype of an isolate and its geographic origin at a national and regional scale [42,45]. These repeated observations indicate that *M*. *ulcerans*, when introduced to an area, remains localized and isolated for a sufficiently long period to allow mutations to become fixed in the bacterial population and a local genotype to evolve. It is reasonable to infer therefore, that the environmental reservoirs of the bacterium in these areas are also likely to be somewhat localized and isolated.

However, the current study has shown for the first time how this focal distribution pattern breaks down at a local scale with the presence of identical genotypes appearing concurrently in separate areas of the same district. There was no discernible distribution pattern for either the Agogo-1 or Agogo-2 genetic clusters, with both *M*. *ulcerans* genotypes appearing at the same times and within the same villages across the region. Interestingly, there were several examples of isolates with identical genome sequences (e.g. isolates F74, 1510 or F85, F65) that were obtained from patients living in four different villages, each separated by distances in excess of 10km (Fig. 2). There are several potential explanations for these patterns. The bacteria (or a vector spreading the bacteria) may be widely distributed across the region and infections are being acquired locally, or it may be that people are traveling and becoming infected from a common point source. Patient interviews and travel histories did not reveal any common activity that might explain a point-source transmission scenario, although the long incubation time for this disease (4-months) is likely to make recall of any such events unreliable [46]. However, on balance the former scenario seems most likely, and we suggest that each genotype of *M*. *ulcerans* has now spread equally widely across the region. If this assumption is correct, then the lack of genetic variation among isolates suggests that the spread of *M*. *ulcerans* throughout the region has occurred relatively rapidly, with insufficient time elapsed for mutations to accumulate. Reliable mutation rates for *M*. *ulcerans* have not been established and some solid data here would allow inferences regarding the time particular clones have been extant within a population.

To our knowledge, this is the first report to employ whole genome sequencing to explore the molecular epidemiology of BU at a local scale. A previous study utilizing high-resolution SNP assays to explore *M*. *ulcerans* genetic variation did uncover some suggestion of local genotype clustering and a recent report used VNTR to examine the link between human and environmental sources of *M*. *ulcerans* [30,47]. However such approaches rely on variable nucleotides that have been defined from a limited reference genome set. If this reference genome set does not represent the genetic variation of the isolates under investigation then data analysis can be flawed, with phenomena such as long-branch attraction and phylogenetic discovery bias confounding analyses [48]. Whole genome sequencing and comparisons of all isolates under investigation as in our study here overcomes the potential weaknesses of targeted SNP-based typing. SNP-typing could however be employed to classify patient samples as Agogo-1 and Agogo-2 genotypes without relying on sequencing of cultured isolates, as culture sensitivity is only around 30%, depending on transport duration. Future studies could thus search for clinical phenotypes between these two distinct bacterial genotypes, although no differences were observed in pathology or treatment outcomes among the patients associated with this study.

There are interesting parallels between *M*. *ulcerans* and *Mycobacterium leprae*, the causative agent of leprosy, where genomics has shown that the leprosy bacillus is another example of a niche-adapted, highly clonal, zoonotic mycobacterial pathogen, with the potential to spread from environment-to-human [49–52]. *Mycobacterium tuberculosis* might also be considered in a similar context, with genomic population analysis also suggesting interactions among genetically distinct *M*. *tuberculosis* lineages [53,54].

One potential issue arising from this study is the risk of incorrectly attributing Nigeria as the origin *M*. *ulcerans* genome sequence ITM102686, as it represents only one isolate. While the patient history makes a persuasive argument for Ibadan as the source of the infection, additional *M*. *ulcerans* isolates are clearly required from patients in different BU endemic regions of Nigeria and surrounding countries, to further explore the relationship and disease transmission patterns we propose here.

Regardless of the precise origin of Agogo-2 isolates, the data presented here suggest that *M*. *ulcerans* can be introduced into a region and then be spread extensively. How might *M*. *ulcerans* be imported into a region? We speculate that movements of people or perhaps animals between countries could be one likely means, where infected individuals with BU lesions that can contain very high bacterial burdens might inadvertently contaminate aquatic environments during bathing or other water contact activities. Now is the time to undertake more intensive and extensive whole-genome *M*. *ulcerans* sequencing surveys across West Africa, to assess the extent of genotype admixture such as we’ve revealed here. Enriching our genome data will also inform other research programs that are identifying reservoirs of *M*. *ulcerans*, leading to the new knowledge required to design interventions and stop the spread of BU.

## Supporting Information

S1 Table Summary of the 127 variable nucleotides that differentiate the Agogo-1 and Agogo-2 clusters, with predicted CDS consequences, based on whole genome sequence comparisons. (XLSX)
