# Correction: Whole Genome Comparisons Suggest Random Distribution of *Mycobacterium ulcerans* Genotypes in a Buruli Ulcer Endemic Region of Ghana

**DOI:** 10.1371/journal.pntd.0003798

**Published:** 2015-05-13

**Authors:** Anthony S. Ablordey, Koen Vandelannoote, Isaac A. Frimpong, Evans K. Ahortor, Nana Ama Amissah, Miriam Eddyani, Lies Durnez, Françoise Portaels, Bouke C. de Jong, Herwig Leirs, Jessica L. Porter, Kirstie M. Mangas, Margaret M. C. Lam, Andrew Buultjens, Torsten Seemann, Nicholas J. Tobias, Timothy P. Stinear

There is an error in Table 1. The values in the “ENA” column are missing. Please see the corrected Table 1 here.

**Table 1:** *M*. *ulcerans* isolate and DNA sequencing information

No.	Strain ID^1^	Isolation date	Patient Age(years)	Patient Gender	Village/Genotype	District/Country	Location^2^	Seq Plat.	Avg cov.	ENA	Ref.
1	S15	02/02/2010	5	M	Ananekrom/ Agogo-1	Ashanti/Ghana	6.91481, -1.01658	SE PGM	106	SAMEA3212578	This study
2	S43	09/05/2010	13	F	Ananekrom Agogo-2	Ashanti /Ghana	6.91481, -1.01658	SE PGM	120	SAMEA3212570	This study
3	S38	23/06/2010	12	M	Serebuso/ Agogo-1	Ashanti /Ghana	6.94838, -1.02373	SE PGM	104	SAMEA3212580	This study
4	F64	08/09/2010	3	M	Nsonyameye/ Agogo-1	Ashanti /Ghana	6.93744, -0.96464	SE PGM	99	SAMEA3212574	This study
5	F70	15/09/2010	32	F	Baama/ Agogo-1	Ashanti /Ghana	6.96810, -0.94013	SE PGM	61	SAMEA3212582	This study
6	1510 (F79)	22/09/2010	28	F	Bebuso/ Agogo-2	Ashanti /Ghana	6.88909, -0.97209	PE Illumina	81	SAMEA3212565	This study
7	F75	07/10/2010	18	F	Afriserie OK/ Agogo-1	Ashanti /Ghana	7.01992, -0.92325	SE PGM	68	SAMEA3212581	This study
8	F85	20/10/2010	28	F	Nshyieso/ Agogo-2	Ashanti /Ghana	6.95910, -1.01860	SE PGM	71	SAMEA3212566	This study
9	S77	24/11/2010	3	F	Serebuso/ Agogo-2	Ashanti /Ghana	6.94838, -1.02373	SE PGM	83	SAMEA3212571	This study
10	2610 (F92)	24/12/2010	14	F	Nsonyameye/ Agogo-1	Ashanti /Ghana	6.93744, -0.96464	PE Illumina	67	SAMEA3212567	This study
11	F13	02/02/2011	8	F	Bebosu Ado/ Agogo-1	Ashanti /Ghana	6.88909, -0.97209	SE PGM	92	SAMEA3212583	This study
12	F65	02/09/2011	28	F	Ananekrom/ Agogo-2	Ashanti /Ghana	6.91481, -1.01658	SE PGM	89	SAMEA3212576	This study
13	F74	21/09/2011	38	M	Dukusen Agogo-2	Ashanti /Ghana	6.97683, -0.98278	SE PGM	46	SAMEA3212572	This study
14	S72	12/12/2011	11	M	Afriserie/ Agogo-1	Ashanti /Ghana	7.01992, -0.92325	SE PGM	128	SAMEA3212575	This study
15	612 (F36)	18/07/2012	37	M	Ananekrom/ Agogo-1	Ashanti /Ghana	6.91481, -1.01658	PE Illumina	97	SAMEA3212568	This study
16	212 (F3)	08/02/2012	11	F	Serebuso/ Agogo-2	Ashanti /Ghana	6.94838, -1.02373	PE Illumina	62	SAMEA3212577	This study
17	712 (S24)	02/08/2012	40	F	Wenamda/ Agogo-2	Volta/Ghana	7.07738, 0.092016	PE Illumina	214	SAMEA3212569	This study
18	412 (F37)	02/08/2012	12	M	Ananekrom/ Agogo-1	Ashanti /Ghana	6.91481, -1.01658	PE Illumina	162	SAMEA3212584	This study
19	IC21	07/2000			Bondoukou	Cote d’Ivoire	8.03333, -2.8000	SE PGM	79	SAMEA3212579	This study
20	IC38	07/2000			Dimbokro	Cote d’Ivoire	6.64445, -4.70540	SE PGM	63	SAMEA3212573	This study
21	ITM000909	2000			Tchekpo Deve	Maritime/Togo	6.48434, 1.369718	SE PGM	45		[45]
22	ITM991591	1999			Anagali	Maritime/Togo	6.48434, 1.369718	SE PGM	55		[45]
23	ITM102686	2010		M	Ibadan	Oyo State/Nigeria	7.50194, 3.982327	SE PGM	51		[45]
24	ITM5151	1971				Maniema/DRC^3^	-4.18655, 26.43937	SE PGM	51		[45]
25	NM14.01	2001				Densu/Ghana	5.72532, -0.31075	PE Illumina	111		[42]
26	NM43.02	2002				Densu/Ghana	5.69881, -0.38597	PE Illumina	112		[42]
27	NM49.02	2002				Densu/Ghana	5.70209, -0.29818	PE Illumina	114		[42]
28	NM54.02	2002				Densu/Ghana	5.6568, -0.32419	PE Illumina	73		[42]
29	NM33.04	2004				Amansie West/Ghana	6.68712, -1.62197	PE Illumina	84		[42]
30	Agy99^4^	08/1999	5	F	ulcer	Amansie West/Ghana	6.68712, -1.62197	Sanger			[41]
31	ITM980535	1998	12	F	Djigbé	Atlantique/Benin	6.885076, 2.362017	PE Illumina	257		[42]
32	ITM000945	2000	21	F	Hwegoudo	Atlantique/Benin	6.7234, 2.377314	PE Illumina	231		[42]
33	ITM001506	2000	6	F	Wokon	Zou/Benin	7.099851, 2.464233	PE Illumina	233		[42]

Notes:

^1^parentheses indicate alternate strain code,

^2^expressed as latitude and longitude,

^3^Democratic Republic of the Congo,

^4^reference genome
